# Diffuse PTH expression in parathyroid tumors argues against important functional tumor subclones

**DOI:** 10.1530/EJE-15-1062

**Published:** 2016-05

**Authors:** Felix Haglund, C Christofer Juhlin, Nimrod B Kiss, Catharina Larsson, Inga-Lena Nilsson, Anders Höög

**Affiliations:** ^1^ Department of Oncology-PathologyKarolinska Institutet, Karolinska University Hospital CCK, SE-171 76 Stockholm, Sweden; ^2^ Department of Molecular Medicine and SurgeryKarolinska Institutet, Karolinska University Hospital CCK, SE-171 76 Stockholm, Sweden

## Abstract

**Objective:**

Primary hyperparathyroidism is usually characterized by a monoclonal parathyroid tumor secreting excess parathyroid hormone (PTH). The main regulator of PTH secretion is calcium and the calcium–PTH set point is shifted in parathyroid tumor cells. We sought to investigate the relationship between tumor PTH and *PTH* mRNA expression and clinical presentation as well as the regulatory factors including phosphate, vitamin D, and fibroblast growth factor 23.

**Design:**

A total of 154 parathyroid tumors were analyzed by PTH immunohistochemistry and chromogenic *in situ* hybridization of *PTH* mRNA. A subset of samples (*n* = 34) was analyzed using quantitative real-time PCR.

**Results:**

Low tumor *PTH* mRNA level was significantly associated with low tumor PTH immunoreactivity (*P* = 0.026), but the two did not correlate with regard to histological distribution within individual tumors. Tumors displaying reduced *PTH* mRNA levels as compared with normal rim were significantly larger (*P* = 0.013) and showed higher expression of the *calcium-s*ensing *receptor* (*CASR*) (*P* = 0.046). Weaker tumor *PTH* mRNA level was significantly associated with higher concentration of circulating 25-hydroxyvitamin D (*P* = 0.005). No significant correlation was seen between PTH immunoreactivity and patient biochemistry. Tumor weight was strongly associated with circulatory concentrations of calcium and PTH.

**Conclusions:**

No areas with apparently higher PTH expression were identified, perhaps suggesting that hyper functioning parathyroid tumor subclones should be rare. Circulating 25-hydroxyvitamin D levels may influence tumor *PTH* expression *in vivo*. If PTH immunoreactivity reflects the tumor calcium–PTH set point, our data imply that the main determinant of disease severity should be tumor weight.

## Introduction

Primary hyperparathyroidism (pHPT) is caused by parathyroid tumors, commonly a parathyroid adenoma (85–90%), or multiglandular disease/primary hyperplasia (10–15%), and rarely a parathyroid carcinoma (<1%) (1). The tumors secrete excess levels of parathyroid hormone (PTH) into the bloodstream, resulting in hypercalcemia. While disease-related mortality is low, multiple studies report increased morbidity (2, 3, 4).

The mechanisms underlying parathyroid tumor formation in the parathyroid glands are incompletely understood. Most parathyroid tumors are believed to be of monoclonal origin (5). Parathyroid adenomas frequently harbor mutations in, or loss of heterozygosity (LOH) of the multiple endocrine neoplasia type 1 (*MEN1*) gene; and parathyroid carcinomas often show mutations in or LOH of the cell division cycle 73 (*CDC73*) gene (6, 7, 8). Overall, whole-exome sequencing studies have found few somatic alterations, with just a limited number of reoccurring genes in the parathyroid adenomas (9, 10, 11).

The chief regulator of PTH secretion is serum calcium, mediated by the G-protein-coupled, membrane-bound calcium-sensing receptor (CASR) (12). The calcium–PTH set point is defined as the serum calcium level which corresponds to 50% of the maximum PTH secretion. Calcium sensitivity is reduced in parathyroid tumor cells, i.e. the calcium–PTH set point is rightward shifted, allowing for persistent PTH secretion despite relative hypercalcemia (13). Other systems also regulate the calcium–PTH set point, e.g. inhibitory feedback by the vitamin D receptor (VDR) and fibroblast growth factor 23 (FGF23)/Klotho-axes (14, 15, 16). Additional effects mediated by estrogens and progesterone have been described previously (17, 18).

Parathyroid cells synthesize PTH as a pre-pro hormone and store it in intracellular vesicles after protein cleavage. Decreased extracellular levels of serum calcium lead to lowering of intracellular calcium, resulting in PTH vesicle exocytosis (12). Regulation of *PTH* gene expression is mediated by serum calcium, 1-25-dihydroxyvitamin D (1, 25(OH)_2_D_3_) and phosphate levels (13, 15). Severe 25(OH)D_3_ deficiency may result in secondary HPT, and after prolonged deficiency patients may present with parathyroid tumors (tertiary hyperparathyroidism). Patients with pHPT frequently suffer from 25(OH)D_3_ deficiency, and some studies report an association with disease severity (19, 20, 21).

These regulatory systems control parathyroid physiology acutely by altering PTH secretion, intermediately by *PTH* gene transcription, and in the long-term by parathyroid cell proliferation. Multiple studies suggest that parathyroid tumor proliferation and/or formation are related to aberrations in these regulatory systems in pHPT.

We aimed to investigate the potential relationship between parathyroid tumor PTH secretion, PTH gene expression, and patient clinical parameters in a large, well-characterized cohort of parathyroid tumors. Tumor PTH content was measured by immunohistochemistry (IHC), and PTH gene expression by chromogenic *in situ* hybridization (CISH). 

## Subjects and methods

### Patients and clinical samples

This study included 143 parathyroid adenomas from a prospective cohort operated at the Karolinska University Hospital, and previously described in detail (22, 23, 24). The following clinical variables were included for statistical correlations: patient serum intact PTH, ionized calcium, creatinine, phosphate, 25(OH)D_3_, FGF23, and bone turnover markers: i.e., alkaline phosphatase (ALP), C-terminal cross-linking peptide of type I collagen (CTX), and type 1 procollagen (P1NP). As previously described a standardized protocol for biochemical sampling was used, allowing for accurate comparison between patient and tumor characteristics (22, 23, 24). Furthermore, all tumors were managed by a limited group of specialized personnel at the Pathology Department, ensuring a minimal difference in sample handling. In 89 adenomas, adjacent normal parathyroid tissue (“normal rim”) was present and scored as a separate entity. All samples were collected with informed patient consent and approval of the local ethics committee. The parathyroid carcinomas (*n* = 6) and atypical adenomas (*n* = 5) have been collected on a world-wide basis, and has in part previously been published as part of a historical material (22, 25); reliable biochemical data were not available for these cases. All tumors were classified according to World Health Organization (WHO) 2004 criteria (26) by an experienced endocrine pathologist. Clinical data are summarized in Supplementary Table 1, see section on supplementary data given at the end of this article.

### Immunohistochemistry (IHC)

Tissue sections for IHC and CISH were prepared in direct consecutive cuts. Intracellular PTH levels were assessed using standard immunohistochemistry methodology. In short, formalin-fixated, paraffin-embedded tissue was cut into 4 µm sections and mounted on SuperFrost slides (Thermo Fisher Scientific), followed by manual xylene deparaffinization and alcohol rehydration. As determined by optimization experiments, heat-induced antigen retrieval was done in a microwave oven at 95°C for 20 min using a low pH sodium citrate buffer. Biotin blocking was performed using Avidin-Biotin Kit (Vector Laboratories; Burlingame, CA, USA). Slides were incubated with mouse monoclonal anti-PTH at 1:200 dilution (NCL-PTH-488, Leica Biosystems; Wetzlar, Germany) at 4°C overnight, followed by a horseradish peroxidase-conjugated secondary horse anti-mouse antibody at 1:200 dilution (B-200, Vector Laboratories, Burlingame, CA, USA) for 45 min in room temperature. Immunoreactivity was visualized by avidin–biotin–peroxidase complex method using Vectastain Elite Kit (Vector Laboratories, Burlingame, CA, USA) and 3,3′-diaminobenzidine (DAB) as chromogen. Counterstaining was performed using hematoxylin (Htx). Anonymized samples of normal pancreas and thyroid tissue were used as negative controls, in addition to parathyroid samples processed with omission of the primary antibody.

### Chromogenic *in situ* hybridization (CISH)

Slide preparation and deparaffination were performed as described above. *PTH* mRNA was visualized using a commercially available CISH technique. The protocol is extensively explained in a study by Grabinsky and coworkers (27). We applied the RNAscope 2.0 HD Reagent Kit (cat# 310035) with a probe detecting *PTH* mRNA (cat# 400521), all from Advanced Cell Diagnostics, with few changes to the manufacturer´s protocol. After optimization, pretreatment 2 was lengthened to 15 min and Htx counterstaining time was lengthened to 4 min. In addition, the Bluing solution was omitted; instead, Htx counterstained slides were gently washed in distilled water for 10 min. The same negative controls as for IHC were applied for CISH. In addition, in accordance with the RNAscope protocol, *dapB* (dihydrodipicolinate reductase, cat# 310043, Advanced Cell Diagnostics) was used as a negative control and *PPIB* (Cyclophilin B, included in cat# 310035) was used as a positive control.

### Slide evaluation

All samples were examined by Bright-field microscopy at ×200–400 magnification. A subset of cases was digitalized with a Hamamatsu slide scanner (Hamamatsu, Shizuoka, Japan). After reviewing the intensity for probe staining (CISH) and antibody immunoreactivity (IHC), a semi-quantitative scoring system was developed based on the cytoplasmic staining intensity distribution (both ranging from +1 to +4) (Supplementary Table 2). Thirty cases were randomly selected from the cohort and two pathologists scored them individually and blindly for validation purposes (Cohen’s weighted kappa: CISH 0.813, IHC 0.793). The remaining samples were then scored by a single pathologist. Figures were created by directly exporting images of digitally captured slides, without further processing.

### Quantitative real-time PCR (qRT-PCR)

A subset of cases (*n* = 34) with available RNA was subject to mRNA quantification using qRT-PCR. In short, synthesized cDNA was amplified in triplicates using gene expression MasterMix and TaqMan probes (probes targeting CASR (Hs01047793_m1) and VDR (Hs00172113_m1) mRNAs). Ribosomal protein large P0 (*RPLP0*, Hs99999902_m1) was used as endogenous control all from (Life Technologies). Water and cDNA generated with omitted reverse transcriptase were run in parallel as negative controls. Relative expression levels were calculated by the delta-delta Ct method, samples being normalized against the mean tumor expression value (arbitrarily set to 1).

### Statistical analysis

We used Fisher’s exact test for comparisons between categorical variables. When comparing continuous and categorical variables, Mann–Whitney U-test or Kruskal–Wallis test was used as applicable. Spearman’s rank-order correlation was used for continuous values. All tests were performed as two-tailed, and *P*-values of <0.05 were considered to be statistically significant.

## Results

### PTH expression in parathyroid tumors and normal rim

**Figure 1:**
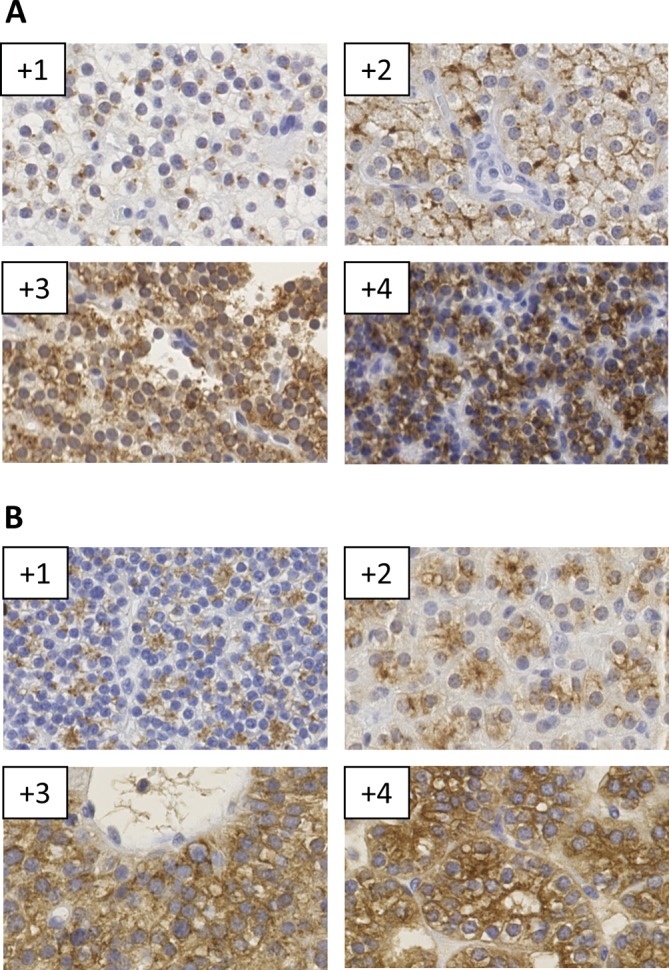
Examples of scoring intensities for (A) PTH immunohistochemistry (IHC), and (B) *PTH* mRNA chromogenic *in situ* hybridization (CISH) of parathyroid tumors.

All parathyroid tumors and normal rims expressed PTH protein and *PTH* mRNA (Table 1). PTH immunoreactivity in the adjacent normal rim was stronger than that in the corresponding tumor cells in the majority of cases (73%; 60/82), the remaining scored equal (26%; 21/82) or weaker (1%; 1/82). *PTH* mRNA levels were also stronger in the normal rim than those in the corresponding tumor cells in the majority of cases (76.5%; 65/85), the rest being equally strong (23.5%; 20/85). The score for staining intensity is illustrated in Fig. 1. Tumors with weak or intermediate *PTH* mRNA levels (+1 to +2) were significantly associated with weak tumor PTH immunoreactivity (+1 to +2) (Fisher’s exact test: *P* = 0.026).

**Table 1:** Tumor and normal rim PTH immunoreactivity (IHC) and *PTH* mRNA (CISH) levels.

	Weak	Intermediate	Strong	Very strong	Tumor (T) vs normal rim (N)		
	(+1)	(+2)	(+3)	(+4)	T > N	T = N	T < N
IHC							
Adenomas	17	58	48	7	1	21	60
Atypical adenomas	0	0	1	3	n.a.	n.a.	n.a.
Carcinomas	0	1	1	4	n.a.	n.a.	n.a.
Normal rim	0	14	46	22	n.a.	n.a.	n.a.
CISH							
Adenomas	11	19	39	67	0	20	65
Atypical adenomas	0	0	2	2	n.a.	n.a.	n.a.
Carcinomas	0	2	1	1	n.a.	n.a.	n.a.
Normal rim	1	2	13	69	n.a.	n.a.	n.a.

T > N, tumor stronger than normal rim; T = N, tumor equal to normal rim; T < N, tumor weaker than normal rim; n.a., not analyzed or not applicable.

**Figure 2:**
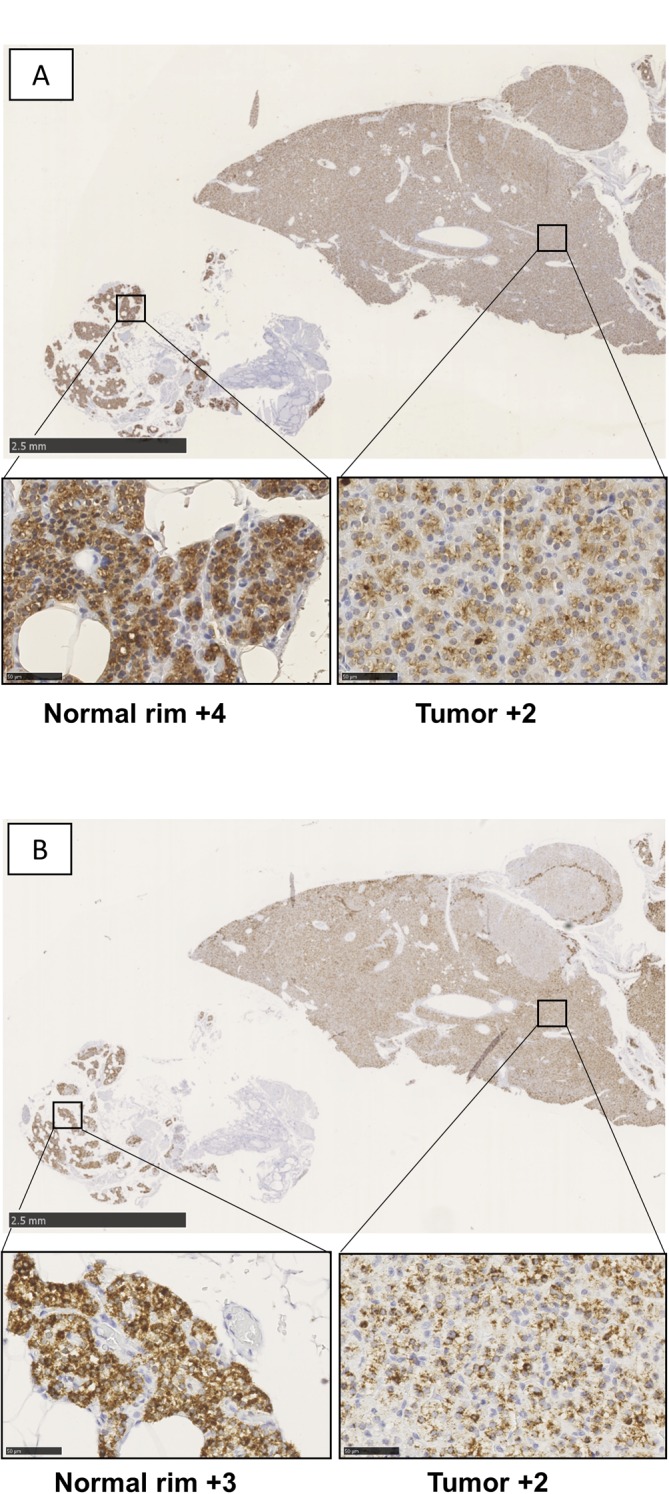
Example of parathyroid adenoma with adjacent normal rim exhibiting tumor heterogeneity and lack of histological correlation between (A) PTH immunohistochemistry (IHC, *left*) and (B) *PTH* mRNA chromogenic *in situ* hybridization (CISH, *right*).

While both PTH immunoreactivity and *PTH* mRNA levels frequently exhibited heterogeneous staining patterns in the tumor tissue, they too did not correlate in terms of spatial distribution within individual tumors (Fig. 2). Tumor cell areas with oxyphilic cell differentiation generally exhibited lower PTH immunoreactivity and *PTH* mRNA levels as compared with adjacent chief cells. However, there was no significant difference between tumors with dominant oxyphilic and chief cell differentiation (data not shown). Individual normal rims exhibited essentially uniform PTH immunoreactivity and *PTH* mRNA staining patterns.

### Clinical correlations of tumor characteristics

Tumors exhibiting lower PTH mRNA levels than the corresponding normal rim were significantly larger (i.e. tumor weight) than tumors with PTH mRNA levels equivalent to their normal rim (Mann–Whitney U: *P* = 0.013; Supplementary Fig. 1A, see section on supplementary data given at the end of this article). To investigate this relationship, a subset of cases with available RNA was analyzed for *CASR* mRNA levels using qRT-PCR. Indeed, tumors with lower *PTH* mRNA levels than the normal rim had significantly higher levels of CASR expression (Mann–Whitney U: *P* = 0.046, Supplementary Fig. 1B). There was no significant correlation between tumor *CASR* mRNA levels and overall tumor weight (Spearman’s rank-order correlation: *R* = 0.299, *P* = 0.085).

Compared with tumors exhibiting strong or very strong *PTH* mRNA levels (+3 to +4), tumors with weak or intermediate (+1 to +2) expression had significantly higher patient serum 25(OH)D_3_ levels (Mann–Whitney U: *P* = 0.005, Supplementary Fig. 1C). While an interesting trend was observed, tumor PTH immunoreactivity (+1 to +4) was not significantly associated with serum circulating 25-hydroxyvitamin D levels (Kruskal–Wallis: *P* = 0.054). Patient serum 25(OH)D_3_ levels correlated significantly weakly with serum circulating PTH levels (Spearman’s rank-order correlation: *R* = –0.195, *P* = 0.026), but did not correlate with tumor weight (Spearman’s rank-order correlation: *P* = 0.133) or ionized calcium (Spearman’s rank-order correlation: *P* = 0.498). In addition, tumor Vitamin D receptor (*VDR*) mRNA levels, as measured by qRT-PCR, were not significantly associated with tumor PTH immunoreactivity or *PTH* mRNA intensity.

Neither PTH immunoreactivity nor *PTH* mRNA levels were significantly associated with patient serum FGF23, PTH, ionized calcium, phosphate levels or bone turnover markers.

Tumor weight significantly correlated with serum ionized calcium concentration (Spearman’s rank-order correlation: *R* = 0.254, *P* = 0.004) and PTH expression (Spearman’s rank-order correlation: *R* = 0.432, *P* < 0.001) and was inversely correlated with serum phosphate concentration (Spearman’s rank-order correlation: *R* = –0.268, *P* = 0.002).

### Parathyroid carcinomas and atypical adenomas had significantly stronger tumor PTH immunoreactivity

All atypical adenomas (*n* = 4) and carcinomas (*n* = 6) investigated expressed PTH and *PTH* mRNA. While the number of cases was limited in this study, the PTH immunoreactivity in atypical adenomas (Fisher’s exact test: *P* = 0.002) and carcinomas (Fisher’s exact test: *P* = 0.003) was more frequently scored as stronger in comparison with parathyroid adenomas.

## Discussion

We observed that a majority of parathyroid adenomas exhibited weaker PTH immunoreactivity and *PTH* mRNA intensity compared with corresponding normal parathyroid rim. The attenuated PTH immunoreactivity plausibly reflects a decreased amount of PTH vesicles within the tumor, and may be explained by decreased PTH mRNA expression, alterations in PTH protein stability and/or reflect the rightward shift in the parathyroid tumor cells’ calcium–PTH set point as a result of increased PTH secretion. While most tumors exhibited some degree of staining heterogeneity, we speculate that this spatial variance is a reflection of biological variation over time in individual cells. This would to some degree explain the lack of spatial correlation between tumor *PTH* mRNA and protein levels. Indeed, tumors with lower *PTH* mRNA levels, as measured by CISH, were significantly associated with lower PTH immunoreactivity, suggesting that reduced PTH expression may partly explain the decreased tumor PTH levels in these cases. We found that a majority of parathyroid tumors had generally lower levels of *PTH* mRNA than the corresponding normal rim. Previously, studies have found both lower and higher *PTH* mRNA expression in parathyroid tumors as compared with normal parathyroids (28, 29, 30, 31, 32). Similar to other studies, we did not observe a relationship between PTH IHC or PTH CISH and serum calcium and serum PTH levels (33, 34). However, a strong correlation was observed between tumor weight and serum PTH and serum calcium levels. If the intensity of tumor PTH immunoreactivity is a reflection of the rightward shift in the calcium–PTH set point, our data indicate that it is the sheer tumor mass, rather than the pathological regulation of PTH, that determines the severity of the disease. While these relationships have been previously presented, this study is the first to integrate both PTH IHC and CISH with patients’ clinical chemistry in a well-characterized large cohort of parathyroid tumors.

Recently, a study by Shi and colleagues has demonstrated that a significant number of parathyroid tumors are of polyclonal origin, with heterogeneous endocrine properties (35). We were unable to distinguish specific areas within individual tumors where PTH immunoreactivity and the *in situ* signal correlated. Our findings are in line with Shi and colleagues, which also reported a lack of correlation between calcium-mediated suppressibility of PTH secretion and disease severity. Taken together, these data argue against a specific entity where a hyperfunctioning tumor subclone is responsible for driving the hyperparathyroidism.

Previous studies have reported downregulation of *VDR* and *CASR* mRNA levels in parathyroid tumors as compared with the normal rim. The level of downregulation was unrelated to patient serum calcium, PTH or 25(OH)D_3_ levels. Carling and coworkers reported a correlation between tumor *VDR* levels and patient serum calcium, but neither this study nor Varshney and coworkers could confirm these findings (32, 36, 37, 38). In our study samples, tumors whose *PTH* mRNA levels were lower than adjacent normal rim were significantly larger than tumors with equivalent levels. These cases also had significantly higher levels of tumor *CASR* expression. In theory, downregulation of CASR levels would allow for a continuously reduced feedback, which would result in both tumor growth and reduced calcium-mediated suppression of *PTH* gene expression. Interestingly, tumor CASR protein levels have previously been coupled to the degree of shift in the calcium–PTH set point, but not to tumor size (13). It is tempting to speculate that this subgroup of tumors has an etiology that is independent of changes in the calcium–PTH set point. Speculatively, a genetic event resulting in parathyroid proliferation while leaving PTH-regulatory systems intact – in this case exemplified by intact *CASR* expression – could account for a phenotype with relatively larger tumor size and lower *PTH* expression per tumor cell. Unfortunately, the lack of a functional cellular model for pHPT currently limits the possibility of functionally testing this hypothesis. Future genetic studies may be able to cluster and correlate genetic aberrations to the level of tumor PTH secretion and clinical phenotype. We observed that tumors with lower *PTH* mRNA levels as measured by CISH had significantly higher levels of circulating 25(OH)D_3_. While the difference was seemingly small (Supplementary Figure 1C) and circulating 25(OH)D_3_ levels did not significantly correlate with PTH immunoreactivity (*P* = 0.054), a mechanism for this relationship has been previously described in *in vitro* settings (39, 40). Some, but not all, studies have reported various correlations between patient vitamin D levels and disease severity (PTH levels, tumor weight, bone turnover). The rationale behind these discrepancies may be due to differences in the study populations, including the degree of vitamin D insufficiency. Specifically, the impact of vitamin D insufficiency may be limited in primary hyperparathyroidism diagnosed at an early stage (18, 19, 20, 21, 32, 40, 41). Indeed, our semi-quantitative immunohistochemistry may be too crude to accurately quantify the tumor PTH levels for weaker statistical relationships. While the value of vitamin D supplementation in pHPT is currently debated, it has been reported to lower serum PTH levels (40, 41). Since our cohort was built up by consecutively collected parathyroid adenomas, it should be the representative of the mild pHPT currently presented in Western populations. Patient circulating serum 25-hydroxyvitamin D levels exhibited a weak correlation with serum PTH level, but were not associated with tumor weight or bone turnover markers (data not shown). While observational, our study suggests that circulating 25(OH)D_3_ levels may influence tumor *PTH* expression in a clinical setting, but further studies are required to assess the possible gains of vitamin D supplementation in pHPT.

## Supplementary data

This is linked to the online version of the paper at http://dx.doi.org/10.1530/EJE-15-1062.

## Declaration of interest

The authors have no conflicts of interest to disclose.

## Funding

This study was supported by Cancer Society in Stockholm, Swedish Cancer Society, Swedish Research Council, Stockholm County Council, and Karolinska Institutet.

### Author contribution statement

FH drafted the manuscript. FH, CJ, NK, CL, I-LN, and AH reviewed the manuscript. FH and NK performed the experiments. FH, CJ, and AH performed slide scoring. FH and I-LN performed statistical analysis. NK and AH designed the study
